# Chinese Public Attention to the Outbreak of Ebola in West Africa: Evidence from the Online Big Data Platform

**DOI:** 10.3390/ijerph13080780

**Published:** 2016-08-03

**Authors:** Kui Liu, Li Li, Tao Jiang, Bin Chen, Zhenggang Jiang, Zhengting Wang, Yongdi Chen, Jianmin Jiang, Hua Gu

**Affiliations:** Zhejiang Provincial Center for Disease Control and Prevention, 3399 Binsheng Road, Binjiang District, Hangzhou 310051, Zhejiang, China; kliu@cdc.zj.cn (K.L.); lli@cdc.zj.cn (L.L.); jiangtao@cdc.zj.cn (T.J.); bchen@cdc.zj.cn (B.C.); zhgjiang@cdc.zj.cn (Z.J.); ztwang@cdc.zj.cn (Z.W.); ydchen@cdc.zj.cn (Y.C.)

**Keywords:** Ebola, Internet surveillance, public attention, Big Data

## Abstract

*Objective*: The outbreak of the Ebola epidemic in West Africa in 2014 exerted enormous global public reaction via the Internet and social media. This study aimed to investigate and evaluate the public reaction to Ebola in China and identify the primitive correlation between possible influence factors caused by the outbreak of Ebola in West Africa and Chinese public attention via Internet surveillance. *Methods*: Baidu Index (BDI) and Sina Micro Index (SMI) were collected from their official websites, and the disease-related data were recorded from the websites of the World Health Organization (WHO), U.S. Centers for Disease Control and Prevention (CDC), and U.S. National Ministries of Health. The average BDI of Internet users in different regions were calculated to identify the public reaction to the Ebola outbreak. Spearman’s rank correlation was used to check the relationship of epidemic trends with BDI and SMI. Additionally, spatio-temporal analysis and autocorrelation analysis were performed to detect the clustered areas with the high attention to the topic of “Ebola”. The related news reports were collected from authoritative websites to identify potential patterns. *Results*: The BDI and the SMI for “Ebola” showed a similar fluctuating trend with a correlation coefficient = 0.9 (*p* < 0.05). The average BDI in Beijing, Tibet, and Shanghai was higher than other cities. However, the disease-related indicators did not identify potential correlation with both indices above. A hotspot area was detected in Tibet by local autocorrelation analysis. The most likely cluster identified by spatiotemporal cluster analysis was in the northeast regions of China with the relative risk (RR) of 2.26 (*p* ≤ 0.01) from 30 July to 14 August in 2014. Qualitative analysis indicated that negative news could lead to a continuous increase of the public’s attention until the appearance of a positive news report. *Conclusions*: Confronted with the risk of cross-border transmission of the infectious disease, online surveillance might be used as an innovative approach to perform public communication and health education through examining the public’s reaction and attitude.

## 1. Introduction

The Ebola epidemic of West Africa had been viewed as a public health emergency of international concern by the World Health Organization (WHO) on 8 August 2014, attributed to its explosive course and high fatality [1]. Ebola virus disease (EVD), also known as Ebola hemorrhagic fever, was first identified in Yambuku and the surrounding areas in Zaire and South Sudan in 1976 [2,3]. The largest outbreak, recently, began in Guinea in December 2013 and had exceeded all of the previous outbreaks combined [2,4,5,6,7,8,9,10,11,12]. The infected initially experienced atypical clinical symptoms of fever, headache, joint/muscle pain, diarrhea, vomiting, and abdominal pain [13,14]. It could be misdiagnosed as some other diseases sharing similar clinical signs, such as malaria or dengue fever [15]. Considering those symptomatic persons evading diagnosis and treatment, the delayed release of laboratory diagnostic data, and the infected person who died undiagnosed, the actual number of the infected could be even higher than announced by WHO [16]. Despite the EVD cases reported in a few countries, the unprecedented concerns had been focused worldwide.

China now has a well-established network to satisfy the increasing demand from netizens. According to the China Internet Development Statistical Report, it claimed a total of 632 million Internet users (46.9% of the whole population) and 527 million mobile Internet users as of 30 June 2014. Meanwhile, the utilization rate of mobile phones (83.4%) first surpassed the traditional personal computer (PC) (80.9%) as one of the commonly used browsing devices. Thanks to extended Wi-Fi coverage, the growing maturity of 3G networks and the enabled 4G networks, Chinese Internet users were online for an average of 25.9 h per week in 2014 [17]. The report also claimed that microblogs and social networking sites had been inevitably shocked by some social applications, such as Wechat. This could be caused by the advantage of the interactivity of Wechat over traditional social media. However, it is undeniable that social media still has its market with user populations accounting for 40.7% of Internet users (nearly 257 million). As the most popular microblog in China, Sina Microblog had the largest number of users, approximately 368 million at the end of 2012 [18]. Furthermore, the Sina Micro Index (SMI), being able to investigate blog data posted and forwarded daily by searching keywords, might mirror the topics focused on by Internet users. On the other hand, searching online serves as one of the most common activities for Internet users to retrieve information. According to the report mentioned above, Baidu is the most popular search engine among Chinese cyber users with a priority selection incidence of 89.1%. The Baidu Index (BDI), stemming from the search frequency on the Baidu search engine, could be employed as a data source for awareness of cyber users. Based on the search volume by netizens on the Baidu search engine, BDI was calculated and exhibited on the basis of special keywords [19]. As a reference value, BDI could comprehensively reflect users focusing on the keywords in the past day and the media attention to these keywords. The China Internet Network Information Centre reported that 97.4% of Chinese netizens chose Baidu as the comprehensive website in the last six months [20]. With the growth of Internet users in China, websites and social media have become vital channels for the public to express their opinions and release their emotions towards major events, including public health emergencies. Therefore, effective and efficient online monitoring is essential not only for the surveillance of public health events at the early stage but also for indispensable interventions to ease the negative public opinions online and to prevent rumors. 

As we all know, the public opinions as expressed through social media are a double-edged sword. Appropriate reactions to the Ebola outbreak in West Africa might enhance individuals’ awareness of protection and hygienic knowledge, whereas overreaction could do a disservice by arousing public panics or spreading rumors among the population. Previous studies used network tools like Tweets or Google to analyze the public concern during an influenza outbreak and perform disease surveillance [21,22,23,24,25,26,27]. In China, BDI, as an effective early indicator, has also been proved to predict the potential cases of epidemic erythromelalgia, and monitor influenza epidemics [28,29]. However, the utilization of Chinese cyber indices, like BDI and SMI, for the purposes of health education was limited. 

Although the Ebola outbreak did not occur in China, it aroused great attention in social media for the potential of imported risk. In our study, we took advantage of BDI and SMI to investigate the Chinese public reaction to the Ebola outbreak in West Africa during the period of tremendous network attention in China. This network-based digital epidemiologic study, on the strength of the Big Data platform, was not only to investigate the public reaction to the “Ebola” emergency but identifying the spatio-temporal distribution and exploring the probable factors associated with the public reaction, which might provide clues for government and health authorities to reform existing modes of health education.

## 2. Materials and Methods

### 2.1. Study Design and Population

#### 2.1.1. Overview

To understand the public reaction in China to the outbreak of EVD in West Africa, we carried out an innovative network digital epidemiologic study based on the online data retrieved from 20 July to 4 September in 2014, in which the epidemics had aroused significant attention and reaction in China.

#### 2.1.2. Data of BDI and SMI, Sources of Ebola Case and Internet Users’ Information

According to the Chinese keyword of “埃博拉 (Ebola)”, the BDI and SMI were collected from the websites of Baidu Index and Sina Micro Index daily, respectively [19,30]. All of the Ebola-related data, including the number of cases and deaths, were collected from the websites of the World Health Organization, Centers for Disease Control and Prevention and National Ministries of Health (USA), and netizen data were from the 33rd Statistical Report on Internet Development in China [31].

### 2.2. Internet Surveillance of Public Reactions and Headline News of Ebola

We initiated Internet surveillance of cyber citizens’ reactions to Ebola from 20 July in China. The daily BDI was recognized as a vital data source, which could provide information involving the weighted sum of search frequency for a keyword in light of its daily search volume via the Baidu website. We gathered the daily BDI with “埃博拉 (Ebola)” as the keyword in cities/provinces to examine the public response. Additionally, Given Internet users in different locations, the average BDI was calculated to identify the mean attention of the netizens (1/100 million) in each province and some cities. Also, we used the same strategy to investigate the blogs posted and forwarded daily for the topic of “Ebola” by the Sina Micro Index (SMI). The headline news reports concerning “Ebola” were also collected. These media events were retrieved from two sources: the headlines by the Baidu Index and Baidu News [32]. The former does not carry news headlines of the same topic every day, especially of topics with minor fluctuations of BDI. The latter not only provides the related media events but also sorts focused news by the topic word. Finally, the collected media events were abstracted and categorized as positive or negative news. Media events were classified as negative if it generated negative sentiments or attitudes towards the topic of Ebola, or as positive if it aroused optimistic sentiments or supportive attitudes towards “Ebola”. These were determined by two individuals, and were eventually decided by the third person if a discrepancy existed.

### 2.3. Statistical Analysis

We graphed the curves of the Ebola outbreak in West Africa to describe the severity of epidemics. To explore the public reaction, the average public reaction of the BDI (average BDI) among Internet users from different regions was calculated by the mean or median (P50), in which P50 was used as data distribution did not suffer the test of normality. Spearman’s rank correlation was employed to check the relationship of epidemic trends with BDI and SMI. The autocorrelation analysis included general autocorrelation analysis and local autocorrelation analysis. The general autocorrelation used the global Moran’s Index. According to the value of Moran’s Index, the result would be determined as a clustered distribution, dispersed distribution, or random distribution, respectively [33]. When the *p* value of the global Moran’s Index was less than 0.05, the local autocorrelation analysis would be carried out by local Getis’s Gi* to identify the potential hotspots. Additionally, Kulldorff’s space-time scan statistics was carried out to recognize the special cities/provinces with high attention to “Ebola” [34]. The parameters of the maximum spatial cluster size and maximum temporal cluster size used the default settings (50%). The log likelihood ratio (LLR) was calculated through comparing the real average BDI with the expected average BDI, and a Monte Carlo test (*p* < 0.05) was utilized to determine the most likely clustered regions. Spatial-temporal analysis was done using ArcGIS software (version 10.1, ESRI Inc., Redlands, CA, USA) and SaTScan software (version 9.1.1, Boston, MA, USA). All of the results were considered statistically significant if *p* < 0.05.

## 3. Results

### 3.1. EVD Epidemic Trend

**Figure 1:**
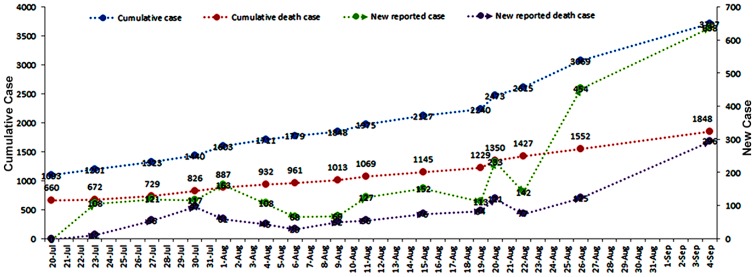
The epidemic characteristics of Ebola outbreak in West Africa from 20 July to 4 September in 2014.

This current Ebola outbreak started in Guéckédou and Macenta districts of Guinea during December 2013 [35], and WHO proclaimed the EVD outbreak on 23 March 2014. As the situation deteriorated, from all of the available evidence, Director-General Margaret Chan of WHO defined the epidemic to be a Public Health Emergency of International Concern. Figure 1 depicts the characteristics of the Ebola outbreak in West Africa between 20 July and 4 September in 2014. 

### 3.2. Daily Baidu Index and Sina Micro Index

**Figure 2:**
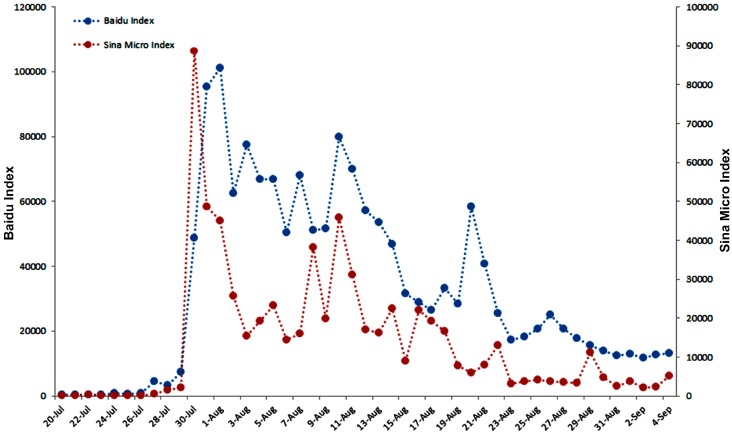
The change trend of the Baidu Index and the Sina Micro Index to the topic of “Ebola” from 20 July to 4 September in 2014.

DBI and SMI were used as the indicators for the public attention to the Ebola outbreak, and the correlation analysis was used to detect the consistency of the two indices (Figure 2). The result showed a positive correlation between BDI and SMI (Spearman’s rank correlation coefficient = 0.9, *p* ≤ 0.05).

### 3.3. Daily Baidu Index for Ebola

#### 3.3.1. Public Attention in the Chinese Mainland to the Ebola Outbreak of West Africa

The BDI for the keyword of “Ebola” increased sharply from 29 July, which peaked at 101,222 on 1 August, and its BDI declined with fluctuations, remaining at a high level above 50,000 between 2 and 9 August. It dropped again on 15 August and reached a lower peak at 79,939. It then steadily decreased to 28,480 with minor fluctuations on 20 August, along with a BDI of 58,360. After that, the BDI of “Ebola” stayed at a lower level between 10,000 and 20,000, but higher than that before 29 July (Figure 2). The data scale ranged from 399 to 101,222, with a median of 25,421 during the study period.

#### 3.3.2. Baidu Index of Available Cities/Provinces

**Figure 3:**
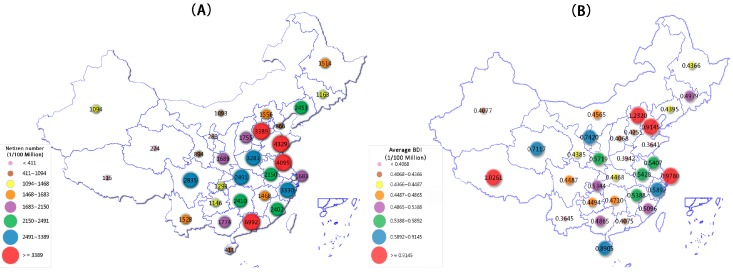
The distribution of Internet users (**A**) and average BDI of “Ebola” (**B**) in Chinese mainland. This map was created by the website of dituhui for free [36].

We further collected the daily BDI of different provinces and some municipalities in China between 20 July and 4 September. The overall trend of the BDI in available cities and provinces was similar. Considering the diverse frequencies among Internet users in different areas, the numbers of netizens were gathered to investigate the average attention as indicated by the average BDI in separated regions (Figure 3). The top five cities/provinces in terms of the average BDI were Beijing, Tibet, Shanghai, Tianjin, and Hainan (Table 1).

#### 3.3.3. Correlation Analysis of Possible Indicators and Public Reaction Online

**Figure 4:**
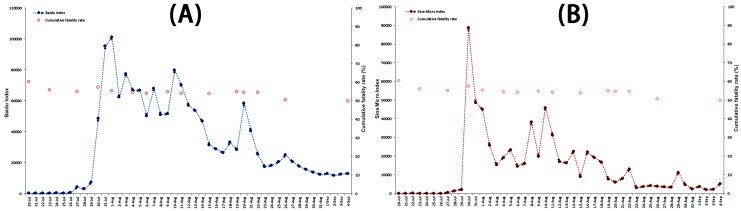
The correlation analysis between the Baidu Index (**A**) and the Sina Micro Index (**B**) of “Ebola” associated with cumulative fatality rate from 20 July to 4 September in 2014.

**Figure 5:**
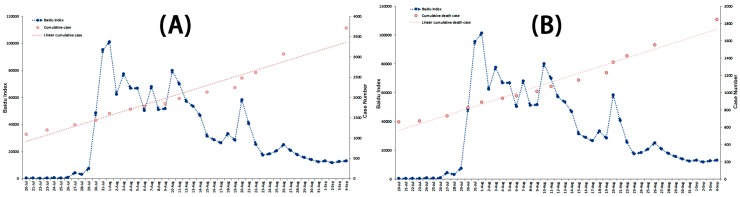
The correlation analysis between the Baidu Index of “Ebola” associated with case-related data from 20 July to 4 September in 2014. (**A**) Association between the Baidu Index and cumulative case; and (**B**) association between the Baidu Index and cumulative death case.

**Figure 6:**
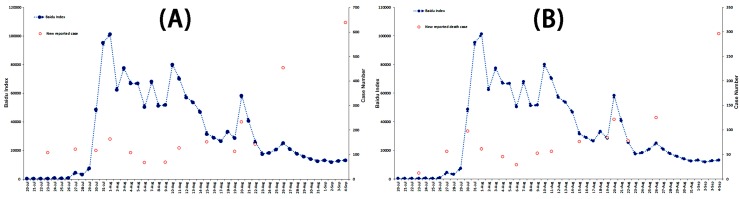
The correlation analysis between the Baidu Index of “Ebola” associated with case-related data from 20 July to 4 September in 2014. (**A**) Association between the Baidu Index and new reported case; and (**B**) association between the Baidu Index and new reported death case.

**Figure 7:**
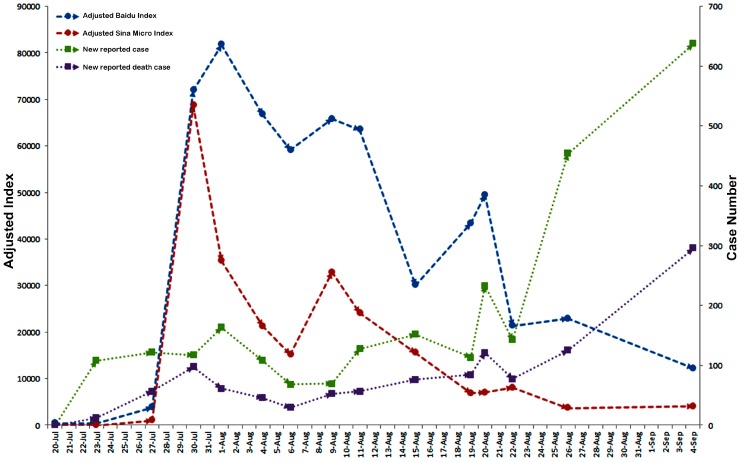
The correlation analysis between the adjusted Baidu Index and the adjusted Sina Micro Index of “Ebola” associated with case-related data from 20 July to 4 September in 2014.

Correlation analysis was carried out to explore potential case-related indicators resulting in the fluctuation of public attention. The associated analyses were performed of the BDI and cumulative fatality rate, BDI and cumulative case, BDI and cumulative death case, BDI and new reported case, and BDI and new reported death case. The results showed no correlation between all case-related influencing indicators and the BDI (Spearman’s rank correlation, *p* > 0.05). We also conducted the correlation analysis of the adjusted BDI and new reported case, and adjusted BDI and new reported death case, which took into account the time difference of America and China (the adjusted BDI being the mean BDI of two adjacent days). No correlation was identified between the adjusted BDI and case-related influencing indicators (Spearman’s rank correlation, *p* > 0.05). These results are detailed in Figure 4A, and Figure 5, Figure 6 and Figure 7.

### 3.4. Microblogs Related to Ebola Posted and Forwarded Daily on the Sina Microblog

#### 3.4.1. Public Attention of the Chinese Mainland to the Ebola Outbreak of West Africa

SMI based on the total microblogs posted and forwarded daily for the keyword “Ebola” on the Sina microblog were also collected. The SMI rapidly increased from 2153 on 29 July to its peak at 88,761 on 30 July 2014, declined to 14,510 with fluctuations on 7 August, and stayed above the primary level before 29 July. The SMI reached another peak at about 45,860 on 11 August, which was lower than the first one, and gradually declined from 31,186 on 12 August to 2056 on 2 September (Figure 2). The data scale ranged from 17 to 88,761 with a median of 7756 during the study period.

#### 3.4.2. Correlation Analysis of Possible Indicator and Public Reaction Online

**Figure 8:**
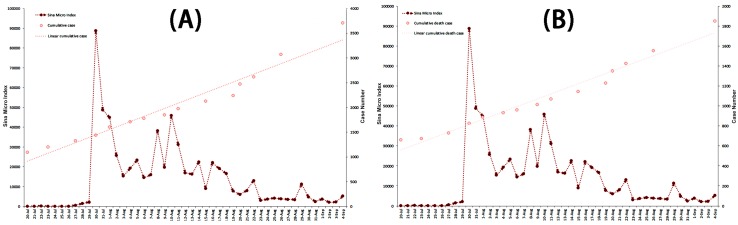
The correlation analysis between the Sina Micro Index of “Ebola” associated with case-related data from 20 July to 4 September in 2014. (**A**) Association between Sina Micro Index and cumulative case; and (**B**) association between the Sina Micro Index and cumulative death case.

**Figure 9:**
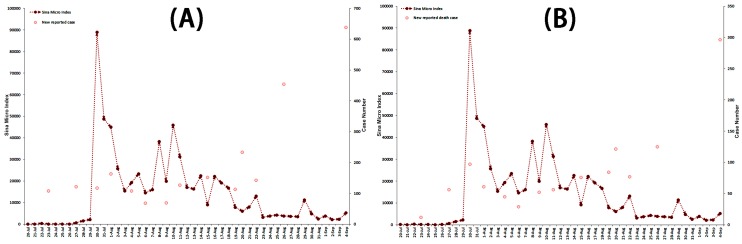
The correlation analysis between the Sina Micro Index of “Ebola” associated with case-related data from 20 July to 4 September 2014. (**A**) Association between the Sina Micro Index and new reported case; and (**B**) association between the Sina Micro Index and new reported death case.

To explore the potential case-related indicator resulting in the fluctuation of public attention, the correlation analysis was carried out. The associated analyses were performed of the SMI and cumulative fatality rate, SMI and cumulative case, SMI and cumulative death case, SMI and new reported case, and SMI and new reported death case. No correlation was found between case-related influencing indicators and the SMI (Spearman’s rank correlation, *p* > 0.05). Correlated analyses were also conducted of the adjusted SMI and new reported case, and the adjusted SMI and new reported death case, which took into account the time difference of America and China (the adjusted SMI being the mean SMI of two adjacent days). No correlation was identified between the adjusted SMI and case-related influencing indicators (Spearman’s rank correlation, *p* > 0.05). The results are detailed in Figure 4B, Figure 7, Figure 8 and Figure 9.

### 3.5. Spatial Autocorrelation Analysis and Spatiotemporal Cluster Analysis

**Figure 10:**
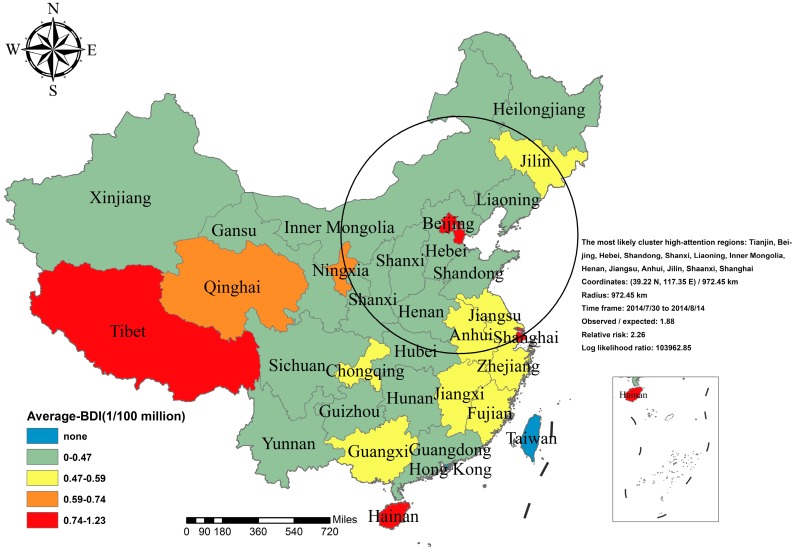
Spatio-temporal clustered characteristics involving the topic of “Ebola” from 20 July to 4 September in 2014.

In the spatial clustering analysis, the general analysis implied that there was significant spatial clustering for the average BDI of “Ebola” in China. The global Moran’s I Index = 0.23 (*p* < 0.01). A local spatial autocorrelation analysis was then performed to identify the hotspot through local Getis’s Gi*. Results of the local autocorrelation analysis showed that the only hotspot to “Ebola” was Tibet. Furthermore, spatio-temporal clustering of public attention to “Ebola” in the study time was carried out. The most likely cluster was identified in the 13 regions of China from 30 July–14 August 2014. The LLR was 103,962.85 with the relative risk (RR) of 2.26 (*p* < 0.01). It included 13 cities/provinces, namely, Tianjin, Beijing, Hebei, Shandong, Shanxi, Liaoning, Inner Mongolia, Henan, Jiangsu, Anhui, Jilin, Shaanxi, and Shanghai. The details are shown in Figure 10.

### 3.6. Qualitative Description of Possible Events during the Study Time

**Figure 11:**
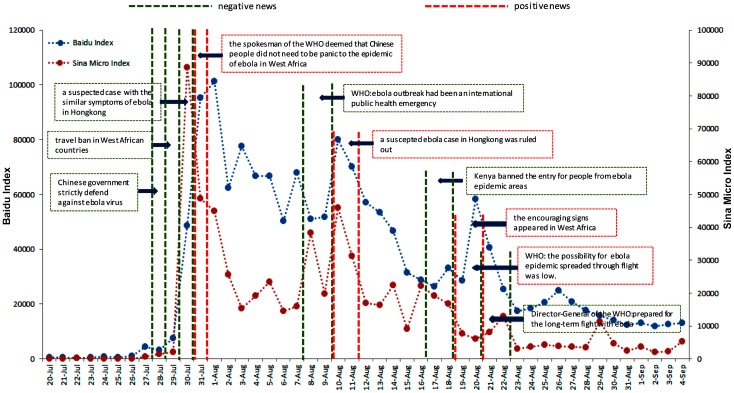
The Chinese news events involving the topic of “Ebola” from 20 July to 4 September in 2014.

As no direct correlation was detected between case-related influencing indicators and BDI/SMI, events possibly related to the fluctuation of public reaction were listed in Figure 11. Our results suggested that a series of negative news reports might cause public concern and nervousness, and subsequently induced a raised public reaction as represented by the network retrieval behavior and the number of microblogs posted and forwarded. A case in point was the report concerning one woman who returned to Hong Kong from Africa with the symptoms of Ebola disease around 30 July in 2014, an event followed by the first peak of the BDI and SMI. Another event was the announcement made around 8 August by WHO, that the Ebola outbreak was identified as an international public health emergency along with the second peak of the BDI/SMI. On the other hand, positive news reports also influenced public attention. When the WHO spokesman deemed that Chinese people did not need to panic for the epidemic of Ebola in West Africa, the BDI/SMI dropped in the next few days from their first peak. Later on, ruling out one suspected case in Hong Kong led to the decline after the second peak. These observations implied that negative news might increase public reaction while positive news might just do the opposite.

## 4. Discussion

### 4.1. Principal Findings

This paper reported the use of BDI and SMI to identify the Chinese public’s reaction to the Ebola outbreak in West Africa from 20 July to 4 September in 2014. Compared with common network tools, including content analysis, indices such as BDI and SMI to investigate public attention possessed unique merits. Firstly, these indicators could identify nearly all retrieved information to the specific keywords on the Big Data platform, while content analysis might only be implemented in limited samples. Additionally, BDI and SMI could mirror the public attention in a timely manner, whereas conventional methods might cause bias, and even be seriously affected by information deletion in websites. In our study, both indices consistently suggested the tremendous public concern to the Ebola event in China. Then, included in the study were the centralized tendency of BDI and average public attention to the Ebola outbreak as indicated by average BDI in different cities/provinces of China. The highest BDI was observed in Guangdong, the province with the largest number of Internet users in China. This might be partly attributed to the opportunities brought about by China’s booming economy, inducing large numbers of West Africans coming to southeast coastal cities including Guangdong, which might lead to overreaction by local netizens. Additionally, the highest average attention to the Ebola outbreak was found in Beijing, the political center of China, along with comparable average BDI in Shanghai, the economic center of the country. The direct flights from these cities to West Africa might contribute to the increase of the average BDI. Interestingly, comparable public attention to Ebola was captured in Tibet, an underdeveloped region, which might be explained by the unique geographical location. Tibet has an underdeveloped transportation system, lower population density, and limited communication, all factors probably contributing to the more frequent web access to acquire information. Therefore, more attention should be paid in Tibet concerning public health education and rumor management. The spatio-temporal analysis had identified 13 clustered regions with higher average attention in China from 30 July to 14 August. During the same period, the daily BDI also indicated higher attention than other periods in China. Thus, we thought that the 15 days after the peak of the BDI was a critical period for infectious diseases with imported risk and that necessary health education intervention should be adopted in these clustered regions. 

Further analyses were performed to explore the correlation between case-related influencing indicators and BDI/SMI. Contrary to our expectation, no existing statistical correlation was established between case-related influencing indicators and BDI/SMI, which was discrepant with our previous findings [18] and other studies [7,37]. This might be attributable to the fact that Ebola epidemics did not occur in China. The public, justifiably, pays more attention to the disease-related data when the outbreak takes place in their location. Otherwise, the public focuses more on the news reports and the notices from the authorities. The assumption was partly testified by the observation that the peaks of BDI and SMI were usually accompanied with some negative news reports and the decline of the indices followed the positive reports. Additionally, the appearance of a suspected Ebola case in Hong Kong might serve as the vital reason for the first peak of BDI and SMI. Moreover, the particular geographic location and administrative position of Hong Kong should be considered as influencing factors of the public’s attention. 

Previous public health experience, such as SARS, indicated that no individual country could single-handedly prevent and protect itself from public health threats. Thanks to the worldwide spread of diseases coupled with the easy access to network information, communicable diseases such as Ebola, did not only affect the locations of the outbreak but also could cause panic or even major public health events in non-endemic areas. In the public health field, increased Internet searching implied the tremendous need of Ebola-related preventive knowledge to the public. That is to say, high attention areas could be more susceptible to rumors or false online information if public health authorities did not address the emerging public concerns in a timely manner. To handle the demand warranted by this emerging situation, traditional epidemiological methods and public health education modes were obviously inadequate. Online surveillance, with the aid of opinion indices, may have sensitively detected the emergence of serious infectious diseases at their initial stage via the Big Data platform in previous studies, which could buy time for controlling outbreaks of these diseases and reducing the risk of transmission to humans [28,38,39]. That is to say, this modality, different from classic epidemiology questionnaires and telephone interviews developed to know the public reaction after the disease outbreak, enables surveillance beforehand and saves health resources. More importantly, online surveillance can reflect public reactions to emergency public health events and disease outbreaks in a timely manner so that public health interventions can be implemented during epidemic crises to avoid deterioration. Additionally, compared to the classical health education mode aimed at high-risk populations in high morbidity regions, our results implied a need for a shift in health education methods to a public-attention-based mode, especially in non-epidemic areas, to identify the regions of high attention. This new mode should be based on the findings of opinion monitoring through public reaction indices like BDI and SMI. Different interventions, in the future, should be adopted for areas with different indices, more attention being targeted at high-index areas in terms of public propaganda and education.

A majority of model studies of the two historical outbreaks of Ebola in the Democratic Republic of Congo and Uganda involved time series analysis [40,41,42]. Further investigation was carried out to assess the dynamics of Ebola in the aspect of the different transmission sources [43]. However, owning to the complicated influencing factors involved in network transmission dynamics, limited research time and medical informatics, an online surveillance model to identify the public concern about Ebola has not been established.

### 4.2. Limitations

Several limitations are mentionable for this study. (1) Considering the deleted Ebola-related blogs, the average SMI was not calculated in our analysis, and the average attention, spatio-temporal distribution characteristics as indicated by SMI in different regions were not verified; (2) The in-depth correlative analysis was not performed because of the absence of clinically characteristic data and detailed distribution information; (3) Our study only focused on Chinese websites and netizens from Chinese mainland, which could not depict the public reaction from Internet users of websites in English or other languages, for instance, Twitter and Facebook; (4) Our findings had not yet been directly applied to identify public health emergency. Meanwhile, these findings had not been evidenced with other search engines; (5) Due to lack of data, we did not analyze the association of the public attention with the migration population from Africa to China and the direct flights from West Africa to China; (6) Although limited adjustment used in our study, the potential time lag was not considered in our study; (7) Traditional media such as TV, newspapers might track well with Ebola case data whereas these were not considered in this study.

## 5. Conclusions

This digital epidemiologic study suggested that online surveillance reflected significant attention in the Chinese population to the Ebola outbreak, and that BDI and SMI were rapid and efficient in identifying and evaluating public reactions. We also identified the regions that paid significant attention to the outbreak. Additionally, compared to domestic outbreaks of epidemic diseases, EVD, which had not occurred in China, might affect the public reaction through positive and negative news reports. In sum, confronted with the risk of cross-border transmission of the infectious disease, online surveillance based on Big Data platforms might be an innovative approach to purposefully perform public communication and health education, which was helpful to avoid the occurrence of public panics and dispel rumors.

## Figures and Tables

**Table 1:** The public reaction of Internet users to the topic of “Ebola” through the BDI.

Province/City	Centralized Tendency *	Netizen Number (1/100 Million)	Average BDI (1/100 Million)
Beijing	1917	1556	1.23
Shanghai	1646	1683	0.98
Guangdong	2849	6992	0.41
Fujian	1224	2402	0.51
Tianjin	792	866	0.91
Zhejiang	1962	3330	0.59
Liaoning	1078	2453	0.44
Jiangsu	2214	4095	0.54
Xinjiang	446	1094	0.41
Shanxi	714	1755	0.41
Qinghai	195	274	0.71
Hebei	1442	3389	0.43
Hainan	366	411	0.89
Shaanxi	966	1689	0.57
Shandong	1576	4329	0.36
Chongqing	691	1293	0.53
Inner Mongolia	499	1093	0.46
Ningxia	210	283	0.74
Hubei	1113	2491	0.45
Jilin	579	1163	0.50
Heilongjiang	661	1514	0.44
Guangxi	863	1774	0.49
Tibet	118	115	1.03
Hunan	1135	2410	0.47
Anhui	1167	2150	0.54
Sichuan	1272	2835	0.45
Henan	1294	3283	0.39
Gansu	392	894	0.44
Guizhou	515	1146	0.45
Yunnan	557	1528	0.36
Jiangxi	791	1468	0.54

* If the available data met a normal distribution (*p* ≥ 0.05: Shapiro-Wilk test), the mean was used; If the available data not met normal distribution (*p* < 0.05: Shapiro-Wilk test), the median (P50) was used.
